# Agency, goal directedness, and the levels of biological organization

**DOI:** 10.1093/biosci/biag030

**Published:** 2026-05-11

**Authors:** Pierrick Bourrat, Andrew B Barron

**Affiliations:** School of Humanities, Macquarie University, North Ryde, NSW 2109, Australia; School of Humanities & Charles Perkins Centre, The University of Sydney, Camperdown, NSW 2006, Australia; School of Natural Sciences, Macquarie University, North Ryde, NSW 2109, Australia

**Keywords:** purpose, teleology, teleonomy, basal cognition, biological agent

## Abstract

Life and evolution often appear purposeful, but goals and purpose are not parts of reductionist sciences (e.g., modern physics, chemistry). This contradiction has been an enduring conundrum. Traditionally, explanations that invoke goal directedness and agency have been treated cautiously, but a new move argues that we should recognize multiple levels of biological organization—cells, tissues, and organisms—as agents with agendas. In the present article, we offer clarity on when and why the concept of goal directedness is applicable in biology. We offer grounds for distinguishing whether something is a candidate for goal directedness, and we consider whether explanations involving goal directedness (teleonomic explanations) are useful. Teleonomic explanations can be briefer and less computationally demanding than algorithmic explanations, and they can help pose hypotheses for how something might function. However, they risk omitting critical details and misdirecting conclusions. Therefore, although there is a place for goal-directed explanations in biology, they should be used with care.

Evolutionary biologists have typically been extremely cautious when claiming that anything is goal directed (Pittendrigh 1958, Mayr 1961, Ayala 1970, Jacob 1977, Kennedy 1992, Ghiselin 1994, Deacon 2011). To claim an organism, an ecosystem, or a cell is goal directed is to claim that it is an agent with an agenda—that is, working toward an objective. Biologists are trained to avoid these kinds of explanations because appeals to goals or intentions introduce a form of agency that does not belong in explanatory frameworks compatible with physics and chemistry. Invoking goals in an explanation is, for them, like placing agents (e.g., homunculi) with their subjectivity in places they do not belong. As Jacques Monod famously claimed: “The scientific attitude implies the postulate of objectivity—that is to say, the fundamental postulate that there is no plan, that there is no intention in the universe” (Hess 1971)). In biology, evolution is not prospective, watchmakers are blind (Jacob 1977, Dawkins 1986), and goals are to be treated with extreme caution.

Although this standard approach continues to be defended (see, e.g., DiFrisco and Gawne 2025), a new move in biology argues that cells, tissues, and organisms are all agents with agendas—not merely acting as if they have goals but genuinely goal-directed agents (Levin and Dennett 2020, Lyon and Cheng 2023, Chis-Ciure and Levin 2025). Some have argued that agency is a missing part of evolutionary theory (Walsh 2015, Sultan et al. 2022, Moczek and Sultan 2023). Others argue that invoking agency provides a different kind of biological agency (Rosslenbroich et al. 2024, Watson 2024). Finally, some argue that we would make more progress understanding development if we acknowledged that the elements involved are agents at multiple levels of biological organization (Levin 2022, 2024, 2025, Newman 2023).

One champion of this new move, Michael Levin, was provocative from the start. He stated, together with Daniel Dennett, “Ever since the cybernetics advances of the 1940s and 1950s, engineers have had a robust practical science of the mechanism with purpose and goal directedness—without mysticism. We suggest biologists catch up” (Levin and Dennett 2020).

Although Levin and Dennett’s (2020) essay is officially framed nonmystically—presenting agency and goal directedness in fully naturalistic terms—its rhetoric sometimes presses beyond a purely heuristic gloss, encouraging a more realist-sounding reading in which goal directedness and agency are genuine features of living systems and not merely an *as if* convenience. In that regard, Dennett’s coauthorship is *prima facie* surprising because his influential stances framework distinguishes predictions made from microphysical facts (the *physical stance*) from those made in terms of function (the *design* or *teleological stance*) and, when explanatorily fruitful, from intentional ascriptions (the *intentional stance*; Dennett 1971, 1989). On Dennett’s view, intentional or goal descriptions are typically licensed by predictive and explanatory payoff rather than by any commitment to intrinsic or irreducible purposes.

Dennett’s deflationary treatment of purposive and intentional descriptions dovetails with a commonplace distinction between teleology (actually having a goal) and teleonomy (acting as if having a goal), although overlapping distinctions can be found much earlier (Dresow and Love 2023). Many biologists insist that terms such as *agent* and *goal* are meant in a strictly teleonomic sense that does not conflict with physics or chemistry. Hearts are said to be *for* pumping blood, bacteria are described as *seeking* nutrients, and genes are said to code *for* traits. But none of these activities involve literal intentions or purposes. Such expressions are metaphorical, describing systems that behave as if they were goal directed.

Despite this distinction, the intended meaning of biologists is not always clear when they use goal-directed language; Levin’s scholarship is a prime example of this. This ambiguity has prompted philosophers to analyze these notions more closely (Wilson 2005, Walsh 2015, Okasha 2018, 2024, Moreno and Peretó 2026). In the present article, we will argue that agency and goal directedness, which we will treat as synonymous in the context of biological systems, have a place and an important role in biological explanations; however, there must be clarity about how, when, and why these concepts should be used.

Before doing so, a few remarks are in order. First, we recognize that, in general, agency and goal directedness can come apart: agency talk is sometimes extended to systems that are largely passive or purely reactive, and some phenomena (e.g., evolutionary trends) might be regarded as teleological even though they do not involve an agent pursuing a goal. Nevertheless, because our focus is on organized biological systems, we will not distinguish these notions. Second, we do not claim that our approach and the distinctions we make are resolutely new, nor that they provide a general solution to the problems associated with teleology in all contexts. Various works beyond those already cited have shaped our thinking (e.g., Nagel 1961, Hull 1974, Wright 1976).

Finally, some philosophical accounts extend teleological or prototeleological notions beyond biology, including to certain abiotic systems or artifacts (e.g., Bedau’s clay crystals or Wright’s lump of quartz or forest fire; see Wright 1976, Bedau 1997, p. 59). Although these cases are philosophically instructive, our focus in the present article is deliberately restricted to biological systems. Nonetheless, we believe that our proposal brings some clarity and will be helpful for the practicing biologists who may feel overwhelmed by the thickness of the concepts involved, the disagreement surrounding them, and the wide range of contexts in which they are used.

To provide this clarity, we make two important distinctions. The first concerns whether the system we are considering (be it gene, cell, tissue, organ, organism, or colony) is a candidate for having a goal (i.e., being teleological). The second distinction concerns whether, for the phenomenon we want to explain (be it development, social organization, or behavior), there is a benefit from the explanation including goal directedness (a teleonomic explanation) irrespective of whether the system has a goal. We can represent these two distinctions in a two-by-two table (table 1) because, in principle, teleonomic explanations might involve systems that are candidates for goal directedness or not. Further, algorithmic explanations might also involve systems that are candidates for goal directedness or, more surprisingly, not. A summary of our approach is presented in box 1 as a diagnostic tool for practicing biologists.

## Is the system a candidate for goal directedness?

Goals and purposes are not parts of modern physics. Therefore, a skeptic might always say that they have no place in scientific explanation (Mayr 1961, Monod 1972, Hull 1974). We do not disagree, but we do note that biologists face some particularly thorny phenomena to explain. There is at least one important distinction between a living biological organism and a soup containing the same lipids, proteins, nucleotides, and other biochemical elements. The biological organism is typically able to maintain its existence and reproduce; the soup does not do that. What explains the difference?

To return to Jacques Monod, in *Chance and Necessity* (Monod 1972, pp. 21–22), he articulated that biology lies in what appears to be an epistemic contradiction: namely, that modern science expunged itself of ultimate goals, but biological organisms have purposes. Some organizational theorists have proposed that the organism exhibits an organizational closure (Varela et al. 1974, Rosen 1991, Deacon 2011, Moreno and Mossio 2015, Montévil and Mossio 2015, Kauffman 2019), whereas the soup does not. For our purpose, we will say that organisms are able to maintain their existence for some time and reproduce and that this capacity, although it is crude, can help us make sense of the confusion surrounding goals and agency. If the entity we are considering has the capacity to maintain its existence, then it is at least a candidate for goal directedness. If it cannot even maintain itself, then it is not a candidate for goal directedness.

Our criterion for being a candidate for goal directedness might seem like a low bar to set, but it avoids having to arbitrate whether a system *is* goal directed. That arbitration is mired in discussions of whether the system is *really* able to represent its environment or represent a goal state, which then splits further into discussion of what is necessary for a representation of a goal (Bekoff and Allen 1995, Tomasello 2022). According to some, only organisms with a nervous system are able to represent. But this is too strong for others (Rosslenbroich 2014). Whether a bacterium moving across a gradient has some sort of representation of its environment is debatable (Fulda 2017). Although the question deserves to be answered, the problem of arbitrating whether a system is goal directed goes far beyond the scope of this article. Our crude distinction, intended as a general heuristic, is enough to help clarify when and why goal directedness is invoked in biology.

A further problem that we will bypass is that goals are always relative to a time horizon. For example, it would be equally legitimate to say that a cheetah’s goal is to catch prey, and the same cheetah’s goal is to produce offspring. But these two goals operate at very different timescales. For our purposes, we will only consider goals that are existential—survival and reproduction.

Whether something is able to maintain its existence and to multiply can be used as a guillotine to separate systems that are candidates for having a goal and those that do not, as we illustrate below (see also the two rows of table 1 for illustrative examples). To test whether something can maintain itself and multiply, we propose the following thought experiment: What would happen if we were to isolate the entity in question and relocate it within its biologically normal environment? How would it fare?

**Table 1.**
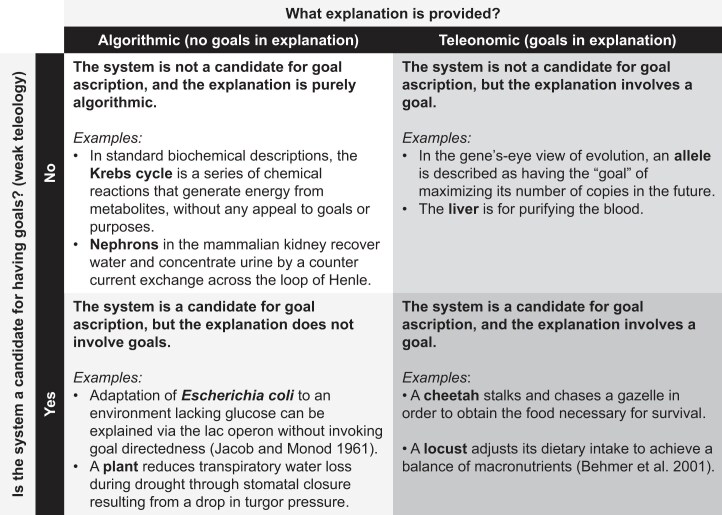
Relationship between a system’s candidate status and mode of explanation.

Before answering, we must clarify what we mean by *biologically normal environment*. We mean the set of components, conditions, and interactions that an entity typically encounters during its functioning—those with which it routinely exchanges material, energy, or information. Any such test inevitably requires selecting which background conditions and interacting factors count as relevant, a choice that is to some degree arbitrary. This parallels the familiar reference-class problem in the philosophy of probability, for which no general solution is available (Reichenbach 1949).

We should also note that while we do not develop the parallel in detail, our test aligns with established criteria for identifying units of selection (Hull 1980, Lloyd 1988, Okasha 2006, Bourrat 2021, 2024)—in particular, with tests for whether an entity counts as an interactor (Hull 1980) or a reproducer (Griesemer 2000, 2014, Bourrat 2025) within the framework of Lloyd (2001, 2024). Our test also bears on McShea’s teleological markers of persistence and plasticity (McShea 2012, Lee and McShea 2020, Babcock and McShea 2021, 2024). Although entities that satisfy our criteria will often display these traits, a full comparison between our approach and that of McShea’s account lies beyond the present scope. More broadly, connecting tests for units of selection with tests for teleological organization may provide new insights in both literatures.

With these remarks in place, consider a human gene (by which we mean a DNA sequence of the human genome coding for a protein; see Griffiths and Stotz 2013). An isolated human gene moved randomly in the nucleus would generally not be replicated because, to be replicated, DNA requires an origin of replication (Kowalski and Eddy, 1989, Blow and Dutta 2005). Therefore, such a gene could only be replicated if it were reintegrated at some specific position within the nucleus of a cell containing the gene replication machinery it needs. Moving the gene out of the nucleus and into the cytoplasm, which might still be regarded as part of its biologically normal environment, would be even more dramatic because, without the replication machinery contained within the nucleus, it could do nothing. Perhaps it would persist for some time, but any damage to it would not be repaired, and it would therefore not maintain its existence. In addition, it could be recognized as potentially foreign DNA by the cell and degraded. This tells us that, according to our test, a gene on its own cannot be a candidate for goal directedness—and therefore a teleological system—even if we confine ourselves to consider the nucleus as the context in which a gene could have an agenda. What this thought experiment aims to discern is whether an entity can only maintain its existence and reproduce if it is integrated as part of a larger system or machine. If so, it is difficult to think of that entity on its own as having the capacity for goal directedness. By contrast, if our entity just needs to be within a system that offers the resources needed for it to survive and reproduce, without having to occupy a specific configuration in the system, then it is more likely to have a capacity for goal directedness. Let us consider some other examples.

What about a human chromosome? An isolated human chromosome would not replicate if it is moved outside the nucleus. Therefore, it is not a teleological system within the context of the cell (Walter et al. 1998). However, there is a limited sense in which it can be a candidate teleological system in the more confined context of the nucleus; if it were reintegrated in the nucleus, it could be successfully replicated because it contains some origins of replication. The gene and chromosome cases can be contrasted with another case, that of retrotransposons.

Retrotransposons are elements of the genome that can be copied from one place of the genome and passed to another place of the genome. The replication mechanism involves transcription into an RNA intermediary, which is then retrotranscribed back to DNA thanks to an enzyme called *retrotranscriptase* (Boeke and Stoye 1997, Wicker et al. 2007). The resulting DNA sequence is inserted in the genome either directly with another enzyme called *integrase* or indirectly for some retrotransposons. In this latter case, the integrase is not involved. Instead, another enzyme called *endonuclease* first nicks the host’s cell DNA, which then permits the retrotranscriptase to start synthesizing the new DNA copy from this point on (Moran et al. 1996, Cost et al. 2002).

There are two types of retrotransposons: autonomous, which encode the proteins for their replication (i.e., retrotranscriptase and integrase or endonuclease), and nonautonomous, which lack this machinery (Kazazian 2004, Wicker et al. 2007). Autonomous retrotransposons can persist and copy themselves in the environment of the cell. In this regard, they are different from genes and chromosomes, which can only be replicated as part of the cell being replicated.

In that sense, autonomous retrotransposons are candidate teleological systems. Nonautonomous retrotransposons can only be replicated if the proteins from other (autonomous) retrotransposons are present in the cell. Therefore, in the absence of these proteins, they are just like a gene or chromosome. In the presence of these proteins, however, they are like autonomous retrotransposons and can therefore be regarded as *bona fide* candidate teleological systems. Overall, the case of retrotransposons is notable because it shows that claims about candidate teleological systems cannot be made in a vacuum: They must be considered relative to a particular environment.

What about a cell? Unicellular organisms are called *free living* for a good reason: they can survive and reproduce just fine if they are moved within their aqueous environments. Even a cell isolated and moved within a multicellular organism could survive for a time and sometimes reproduce. Some cells in multicellular animals are too baroque and specialized to survive for long and cannot reproduce if they are isolated and relocated, such as long motor neurons in large vertebrates (Kandel et al. 2021). But many can. We can grow many cells in vitro on or within a gel. This shows that many cell types can survive very well if they are isolated and moved to a new type of environment. There is therefore some scope to consider cells as a candidate teleological systems *within* the body of an organism and, for some, beyond it. At any rate, within the body of an organism, a cell is a much better candidate for being teleological than a chromosome or a gene.

But what about a tissue? If we were to isolate a part of tissue and move it within the organism, could it survive or even grow? The lesson of skin grafts shows that this can be done, but skin grafts illustrate the need to connect the graft to a sufficient vascular bed to support it (Hosseini et al. 2022). For a tissue graft to succeed, it must be correctly reintegrated into the organism, or it will become necrotic. The demand for reintegration will be less acute if the tissue graft is very small or if the organism is small and less reliant on a vascular system to move resources within the animal. We note the success in reorganizing and grafting tissue in planaria (Lobo et al. 2012), but generally, a tissue is a poor candidate for goal directedness.

Similarly, isolating and moving an organ in its environment would fail in most cases, unless the organ was carefully reconnected to the other organ systems (lymphatic, circulatory, and nervous) needed to sustain it. Much like genes and tissues, in most cases, organs are poor candidates for goal directedness; instead, they are part of something that could be a candidate.

Before moving to the next case, let us consider two potential counterexamples to our claim that tissues and organs are not candidates for teleology: lab-grown meat and organoids. An organoid is a three-dimensional, in vitro–grown structure—typically derived from stem cells—that self-organizes so as to reproduce key cell types and structural (and sometimes functional) features of a real organ (Lancaster and Knoblich 2014). At first glance, both lab-grown meat and organoids might appear to undermine our proposal because they can grow and can be maintained in environments that are not their normal biological environments.

Our test, however, does not assess goal directedness relative to all possible environments. Instead, it first fixes a relevant environment for the entity—a choice that inevitably raises a reference-class problem, discussed above—and then asks whether the entity, when it is placed at random within that environment, would be able to maintain itself as an organized system (and, where applicable, reproduce). For our purposes, we fix the relevant environment of a biological entity as the factors it would normally interact with—what we called its *normal biological environment*—but the test could be applied to different environments.

One *prima facie* way to avoid the need to select a relevant environment would be to appeal to a maximally broad environment shared by all entities. An advantage of this strategy is that it would permit comparisons between any two or more types of entities with respect to their suitability as candidates for teleology. Although eliminating the problem of environment relativity would be a worthwhile theoretical achievement, doing so may ultimately prove infeasible or, at the very least, would require the development of a conceptual apparatus substantially more sophisticated than the one employed here. We therefore adopt a more modest strategy: rather than attempting to eliminate environment relativity altogether, our test proceeds by fixing a relevant environment and assessing goal directedness relative to it, in a manner that reflects the kinds of judgments made in ordinary biological practice.

With this in mind, if we apply our test and fix the relevant environment as a laboratory (rather than an organism), including all the equipment required for growing and maintaining engineered cultures, neither lab-grown meat nor organoids constitute counterexamples. Their viability depends on highly specific conditions, and they would not survive if they were placed indiscriminately within the laboratory environment. In this respect, they are analogous to tissues and organs.

What about a multicellular organism: animal, plant, or fungus? In most cases, an organism can survive and reproduce for a time if it is isolated and moved within its environment without needing to be reintegrated in a particular configuration. Therefore, we can reasonably think that most organisms are candidates for goal directedness.

Finally, many animal societies are also candidates for goal directedness. Many social insect colonies, if they are moved in their entirety in their environment, will prosper very well and continue to develop and reproduce. Honey bee hives are routinely moved in commercial apiculture, and many social ant and bee species are highly mobile naturally, relocating and splitting their colonies to maximize resource exploitation or escape a worsening environment (Hölldobler and Wilson 1994, Oldroyd and Wongsiri 2009).

It is important to emphasize that we are not claiming that cells and organisms are teleological in a *strong* sense. Instead, we argue that some levels of biological organization are reasonable candidates for teleology by virtue of being self sustaining. They are teleological in a *weaker* sense. Returning to the skeptic who might deny that goal directedness and agency have anything to do with physics and chemistry, we respond that the distinction between weak and strong teleology is an instance of a broader distinction between weak and strong emergence (Bedau 1997). A weak sense of teleology captures higher-level phenomena that are reducible, in principle, to lower-level ones, whereas no such reduction is possible for the strong sense of teleology. Strong emergence is treated as mysterious or spooky by most scientists. Via entailment, we believe it is justified to consider strong teleology in the same way as strong emergence and recognize a space and usefulness for a weaker sense that we articulated above. For further examples illustrating our distinction, see table 1 and box 1.

Other levels of biological organization, such as the gene, the chromosome, and tissues, are not self-sustaining; it is difficult to argue for these as candidates for teleology even in the weak sense. The benefits of this distinction become clearer when we consider our second dimension: whether there is benefit from invoking a goal-directed explanation in biology.

## Is there utility in goal-directed explanations?

So far, we have claimed that all higher-level phenomena are, in principle, reducible to lower-level ones. However, this does not mean that all explanations must ultimately be reduced to biomolecular mechanisms or chemistry to be useful. Our only substantive commitment is that any explanation must not violate the laws of physics—a commitment commonly referred to as *materialism* or *physicalism*. To be clear, our position is not that explanations invoking goals enjoy explanatory autonomy in the strong sense of being irreducible, in principle, to mechanistic physical explanations, as was advocated by Walsh (2008, pp. 122–125). Rather, we advance the more modest claim that explanations involving goals can be useful, to the extent that it may be perfectly reasonable to employ them within particular scientific contexts. Our choice originates from the fact that we regard claims of in-principle irreducibility as often serving to smuggle in elements that are incompatible with physicalism. Moreover, as a matter of fact, Walsh (2008) did not successfully defend irreducibility but, instead, advanced a more modest compatibilism between explanations invoking goals and explanations invoking physical mechanisms or processes. We therefore regard our position as compatible in spirit with the view proposed by Babcock and McShea (2023), who appealed to the notion of a field—a physical structure spread out in space, with a value at each point for some property—to bridge mechanistic and goal-directed explanations.

For our purpose, we therefore distinguish two modes of explanation in biology. The *algorithmic* mode describes phenomena in a stepwise, mechanistic way that does not invoke purpose as part of what does the explaining (the structure of the explanation). In contrast, the *teleonomic* mode does invoke purpose as part of what does the explaining (see table 1 for examples of explanations illustrating these two modes). The distinction seems natural if the system is considered a candidate for goal directedness—but much less so if it is not. As we show, teleonomic explanations can be useful even for systems that are not candidates for goal directedness.

We could, for example, provide both algorithmic and teleonomic explanations for the functioning of the heart. An algorithmic explanation of the heart would be a book in length and would include sections on hydrodynamic flow of imperfect fluids through elastic and contractile vessels, the biomolecular mechanisms of cardiac muscle and muscular contraction, and the biomolecular mechanisms of nerves and synapses, among other topics. A teleonomic explanation of the heart is as simple as this: the heart is for pumping blood. This is an obvious shorthand, and it lacks all the detail required of an algorithmic explanation, but there are times when the simple functionality of this teleonomic explanation is all that is needed. Crucially, note that we have established that the heart is an unlikely candidate for goal directedness, but it can figure in a teleonomic explanation.

The classic gene’s eye view of evolution (Hamilton 1964, Williams 1966, Dawkins 1976, 1982, Ågren 2021) is also teleonomic. According to Dawkins, an allele can be seen as an agent trying to maximize its number of copies in a population. The same idea is present in evolutionary game theory (Maynard Smith and Price 1973, Maynard Smith 1982, Hofbauer and Sigmund 1998), in which a mutant strategy can be regarded as if it was an agent trying (and often failing) to maximize its representation in the population. Evolutionary biologists also entertain teleonomic explanations to describe genes as if they controlled phenotypes—and sometimes the phenotypes of other organisms conceived as vehicles for gene propagation. This perspective is particularly present in the context of host manipulation (Dawkins 1976, 1982, Moore and Moore 2002, Poulin 2010, Hughes et al. 2012). Finally, Alan Grafen’s formal Darwinism project seeks to bridge classical adaptationist reasoning with modern mathematical population genetics by showing how natural selection can be represented as if organisms were solving optimization problems that maximize their inclusive fitness—a complex measure of survival and reproduction including that of organisms to which the focal organism is related (Grafen 2007, 2014).

These examples show that there are times where the brevity and functional stance of teleonomic explanations in biology are useful and preferred even in the absence of (weak) teleology in the entities invoked in the explanation.

One might ask what value a teleonomic explanation brings. Biologists generally see teleonomic explanations as dangerous because they might be taken literally and confused with an unscientific teleological explanation (see the references above). We respond the following: generally, an algorithmic explanation will be more precise and detailed—but at the cost of being demanding in both implementation and computation. By contrast, a teleonomic explanation is usually a shorthand. Most teleonomic explanations trade off detail for brevity. In that sense, they are coarse-grained explanations with heuristic and pragmatic advantages over finer-grained algorithmic accounts (Batterman 2001, Bourrat 2023, and the references therein). There are times that this is desirable (consider the example of the algorithmic and teleonomic explanations of the heart given above). The fine detail of an algorithmic explanation constrains it to what is currently experimentally known, up to the point where unknowns limit the explanation.

Acknowledging some advantages of teleonomic explanations must not come at the cost of neglecting two risks that inevitably accompany them. First, these shorthand explanations might not be extrapolable beyond certain conditions (a heart cannot be regarded as if it had a goal outside of an organism). Second, as we mentioned earlier, they might be reified: the *as if* proposition is thought to become genuine. Therefore, teleonomic explanations should be used with great care as glossing over detail and unknowns can steer inference in a seriously wrong direction.

For example, there was a time it was believed that the role of a worker bee was to serve the queen and raise her offspring. This teleonomic explanation of the worker caste masked a far more complex evolutionary dynamic in which the workers collectively control rather than serve the queen (Ratnieks and Reeve 1992). Similarly, the backlash against the form of group selection proposed by Wynne-Edwards (1962) in the 1960s arose from claims that organisms display behaviors or traits for the good of the species or group. Critics argued that such explanations ignored the constraints and dynamics operating at the level of individual organisms (Maynard Smith 1964, Perrins 1964, Williams 1966).

But notice that the very same property of teleonomic explanations—that is, glossing over details and unknowns—makes them incredibly useful for exploring a space of hypotheses. Starting from the idea that an entity has a goal can be fecund. Looking at a structure and thinking of it as an adaptation can help in generating hypotheses for further testing. In this context, biologists have become very good at using *as if* explanations as an exploratory device. If a system operates in such a way, it will display a set of characteristic properties when further tested. The distinction between algorithmic and teleonomic explanations in biology is not merely semantics; it is an important exploratory device.

## Conclusions

Goal directedness and agency have a place in biology but are most useful when used discriminately. We made a distinction between weak and strong teleology as an extension of weak and strong emergence (see table 1 and box 1). The weak sense of teleology captures a system that is a candidate for goal directedness but could also, in principle, be fully explained as mechanisms of physics and chemistry without needing to invoke goal directedness. Strong teleology would capture phenomena that could *only* in principle be explained in terms of goal directedness. The strong sense of teleology sits outside a biological explanation. The weak sense of teleology can be a useful tool if proposing a goal-directed explanation gives us a useful shorthand for how the system might be operating or helps us pose new hypotheses for how the system might function to aid in reaching a more complete algorithmic explanation.

Not everything in biology is a candidate for goal directedness. If a biological entity can only maintain itself and reproduce if it is integrated in a specific configuration as part of a larger unit, then that entity is a poor candidate for goal directedness. It is better considered as a part of a larger machine, where the machine of which it is part might be a candidate for goal directedness. Mistaking goal directedness as a property of a part when the property only belongs to a larger whole is a form of the metonymic fallacy.

Even so, we have provided examples of how we might still apply a teleonomic explanation to entities that are poor candidates for goal directness (see table 1). Physiology texts can describe the liver as for cleansing the blood, and evolutionary texts can describe the goal of a gene as reproduction without committing any mortal sin—as long as there is clear value to the teleonomic explanation. If the teleonomic explanation helps us to explain the entity in a common shorthand or helps us to propose how to understand it in more detail, then it is doing useful work in biological science. If, on the other hand, a teleonomic explanation inadvertently misrepresents how the entity functions or misdirects its further study, then it is counterproductive.

Levin and Dennett (2020) warned us against teleophobia—an irrational fear of or overreaction to goal-directed language in biology. Although we agree in part with their diagnosis, we have shown that indiscriminately ascribing goal directedness to anything and everything—a kind of teleophilia—is at least as counterproductive. Instead, we have shown that there are at least two distinct ways in which goals can be legitimately invoked in teleodiscussions, and that it would be a mistake to collapse them. Moreover, nothing in our analysis suggests that these two roles exhaust the space: There may be further legitimate ways to decompose or refine discussions of goal directedness.

Box 1. **For biologists in a hurry.**

**When is a system a candidate for goal directedness?**

A system is a candidate (weak) teleological system if, when placed in an appropriate environment (see main text), it can maintain its own organization and reproduce for some time. If it can only maintain its existence and reproduce as a part of a larger machine (e.g., as a gene, tissue, or organ that must be embedded in an organism), then any goal directedness is better ascribed to that larger system.

**Two kinds of explanation**.

Algorithmic explanations describe phenomena in stepwise, mechanistic terms without invoking goals or purposes as part of what does the explaining. *Teleonomic explanations* deliberately use goal or function talk (*in order to*, *for the purpose of*) as a compact way of describing how a system tends to behave, while remaining compatible with an underlying mechanistic (algorithmic) account.

**A 2 × 2 space of explanations**.

Crossing these two distinctions (see table 1)—whether the system is a candidate for goal directedness and whether the explanation itself invokes goals—yields four cases. We can have purely algorithmic explanations of systems that are not candidates for goal directedness (e.g., standard biochemical pathways), and teleonomic shorthands applied to such noncandidate systems (e.g., describing genes as having the goal of making copies). We can also have purely algorithmic explanations of systems that *are* candidates for goal directedness (e.g., mechanistic accounts of bacterial gene regulation or plant drought responses), and teleonomic explanations of candidate systems (e.g., a cheetah hunting *in order to* obtain food). Much of the confusion about goal directedness and purpose in biology comes from sliding between these quadrants without noticing.

## Author contributions

PB and ABB both made substantial and essential contributions to the conception, development, and writing of the manuscript. Both authors participated in the formulation of the central ideas, drafting of the text, and critical revision of the manuscript. PB took a leading role in the conceptualization of the work and manuscript preparation. Both authors approved the final version of the manuscript and agree to be accountable for all aspects of the work.
